# ‘Activity-silent’ working memory in prefrontal cortex: a dynamic coding framework

**DOI:** 10.1016/j.tics.2015.05.004

**Published:** 2015-07

**Authors:** Mark G. Stokes

**Affiliations:** Oxford Centre for Human Brain Activity, University of Oxford, Oxford, UK

## Abstract

Working memory (WM) provides the functional backbone to high-level cognition. Maintenance in WM is often assumed to depend on the stationary persistence of neural activity patterns that represent memory content. However, accumulating evidence suggests that persistent delay activity does not always accompany WM maintenance but instead seems to wax and wane as a function of the current task relevance of memoranda. Furthermore, new methods for measuring and analysing population-level patterns show that activity states are highly dynamic. At first glance, these dynamics seem at odds with the very nature of WM. How can we keep a stable thought in mind while brain activity is constantly changing? This review considers how neural dynamics might be functionally important for WM maintenance.

## Highlights

• WM is thought to depend on persistent maintenance of stationary activity states.
• However, population-level analyses reveal that brain activity is highly dynamic.
• Accumulating evidence implicates activity-silent neural states for WM.
• Dynamic coding suggests that WM is encoded in patterns of functional connectivity.

## Searching for the WM engram in the prefrontal cortex (PFC)

There is little doubt that the PFC is important for WM [1]. Some of the earliest studies in nonhuman primates demonstrated that the structural [2] and functional [3] integrity of the PFC is necessary for successful maintenance of information over short delays. Although other brain areas presumably contribute some aspects of WM [4,5], especially modality-specific sensory areas [6,7], the lesion evidence clearly implicates the PFC as a crucial hub necessary for successful maintenance (cf. visual cortex for encoding visual memoranda [8]). This review focuses on neurophysiological recordings in nonhuman primates to disentangle the functional principles of the PFC that mediate WM.

**Figure 1:**
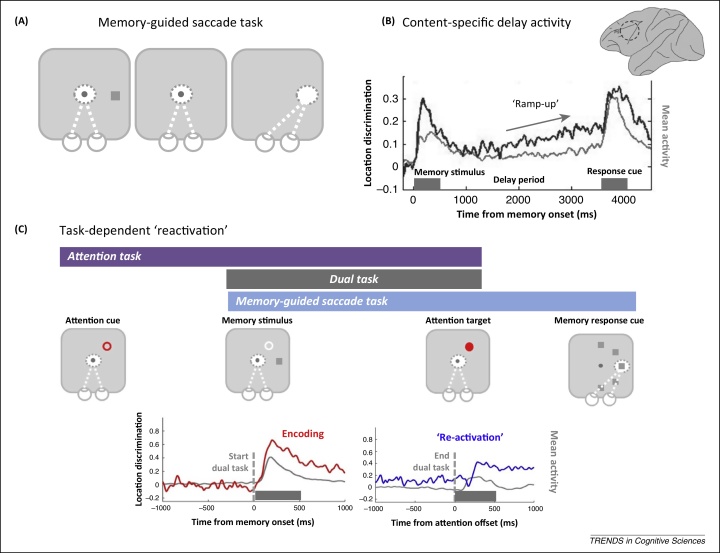
Working memory (WM) delay activity varies with task relevance. **(A)** Schematic of the influential memory-guided saccade (MGS) task (e.g., [1]). On each trial, the subject is presented with a memory location and, after a delay period, executes a MGS. **(B)** Neurophysiological recordings from monkey prefrontal cortex [near the principal sulcus (PS)] during the delay period reveal content-specific activity (location-specific activity is shown in black, population mean activity is shown in grey for reference). However, content-specific delay activity also varies with the current task relevance of the memory item, resulting in a ‘ramp up’ in anticipation of the response. Adapted from [19], with permission from Oxford University Press. **(C)** A more recent dual-task experiment partially overlaid an attention task and the standard MGS design. Although initial encoding of the saccade location was robust (red trace, mean activity shown in grey), content-specific activity was effectively abolished by the dual task during the memory delay (blue trace, from −1000 to onset of the attention target). Critically, location-specific information was ‘reactivated’ at the end of the dual task (blue trace, after onset of the attention target), presumably reflecting a shift in task focus to the MGS. Adapted from [22] with permission from Nature Publishing Group. Such evidence suggests that delay activity reflects the task relevance of memoranda. Gaps in content-specific activity further suggest that WM could be maintained in an ‘activity-silent’ neural state.

Early recordings around the principal sulcus in the monkey PFC discovered an apparent neurophysiological correlate of the WM engram: task-specific delay activity [9,10]. For example, in a delayed reaching task some prefrontal cells increased firing during the delay period, indeed remained active for the duration that response-related information needed to be held in mind, and returned to baseline after the response was executed [9]. A similar sustained firing pattern is observed in a memory-guided saccade (MGS) task [11]. In this now-classic WM task, the subject is presented with a spatial cue at the beginning of the trial that indicates the target for a saccade that can be executed only after a memory delay of a few seconds (Figure 1A). Activity in this memory delay is selective for the specific saccade location, corresponding to the contents of WM (Figure 1B).

Prefrontal delay activity is not limited to motor preparation, but also responds selectively to visual memoranda [12]. Similar to the delayed response tasks, PFC activity during visual delay match-to-sample tasks also covaries with the duration of maintenance in WM. Moreover, some cells even maintain their mnemonic role after the presentation of distracting items, indicating a potential correlate of distracter-resistant WM [12]. Evidence for persistent content-specific delay activity has motivated numerous theoretical [1,13,14] and computational [15,16] models based on the idea that representations are kept ‘online’ after stimulus offset in a stable activity state.

## ‘Activity-silent’ WM

However, maintenance in WM is not always accompanied by an unbroken chain of persistent delay activity in the PFC [17,18]. For example, when the duration of the maintenance period is fixed from trial to trial, robust delay activity sometimes emerges only late during the delay period (Figure 1B) [19] (see also [20]). It seems that when the temporal structure of the task can be predicted, neural activity is reserved for the most relevant time point: that is, preparation for the expected memory probe. So-called ramp-up activity implies that delay activity could optimise WM-guided behaviour by preparing for the processing demands of the behavioural response (e.g., temporal orienting of attention [21]). However, the relatively silent moments between encoding and response preparation additionally suggest that the continuity of vigorous delay activity is not always necessary for the continuity of the mental representation [20].

A recent neurophysiological study reported further evidence suggesting that persistent delay activity might not be necessary for accurate WM-guided behaviour [22]. Critically, this study added a dual task demand to the classic MGS task [1]. As in the standard variant, monkeys were required to encode and maintain the location of a saccade target for execution at the end of the trial. However, they were additionally required to attend to a specific spatial location until part way through the WM delay period (Figure 1C). This cognitive manipulation effectively abolished WM-specific delay activity during the dual task period, even on trials in which WM performance was preserved (WM-correct trials). Moreover, when the competing task demands were completed, content-specific delay activity was ‘reawakened’, presumably in time for WM-guided behaviour. Consistent with the evidence from ramp up, these findings suggest that mnemonic delay activity in the PFC is not always critical for maintaining the continuity of WM but can be dynamically re-established when attention is refocused to the task-relevant content. WM does not seem to depend on delay activity but might be maintained in an ‘activity-silent’ format such as functional connectivity.

## Activity-silent WM stored in functional connectivity

**Figure 2:**
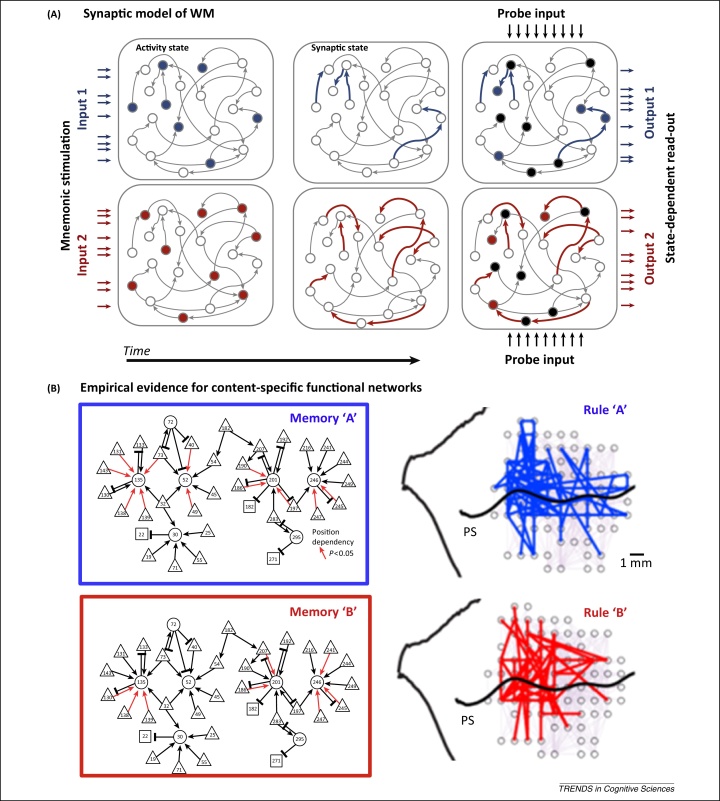
Maintaining ‘activity-silent’ working memory (WM) in functional connectivity. **(A)** Schematic of the synaptic model of WM described in [23]. Task-relevant input (left-side horizontal arrows, blue for ‘Memory A’ and red for ‘Memory B’) drives a stimulus-specific activity state (filled circles) that in turn triggers a specific pattern of short-term synaptic plasticity between cells (bold arrows). Memory is read out from this synaptic trace via the context-dependent response at retrieval (black filled circles, same for ‘Memory A’ and ‘Memory B’). The probe-driven response will be patterned by the hidden state of synaptic efficacy, resulting in a discriminable output pattern (right-side horizontal arrows). **(B)** Empirical evidence for content-specific functional networks. Simultaneous recordings in rat frontal cortex revealed direction-specific patterns of synaptic efficacy (red arrows) between cells [putative pyramidal (triangles); putative interneuron (circle); unclassified (square)], encoding direction during a WM-based maze task (left panel; adapted from [34] with permission from Nature Publishing Group). This is consistent with a role for short-term synaptic plasticity in WM. In the monkey prefrontal cortex (PFC) (PS, principal sulcus), different task rules are associated with specific functional networks (synchronised electrodes for rule ‘A’ in blue, rule ‘B’ in red) coupled by synchrony at ∼30 Hz (left panel; adapted from [36]). This is consistent with the idea that coherence could also play a role in constructing functional networks for flexible behaviour.

According to a synaptic model of WM, information can be maintained during such activity-silent periods as a pattern of synaptic weights [23,24], analogous to long-term memory. In this computational model (Figure 2A), activity during encoding temporarily changes synaptic efficacy within the neural network, leaving behind a temporary synaptic memory trace via activity-dependent short-term synaptic plasticity (STSP) [25]. More specifically, calcium kinetics (see Glossary) provide a window of approximately 2 s for STSP in the PFC [23]; however, presumably different time constants could underlie the diversity of mnemonic timescales observed in the PFC [26]. The essential point is that STSP changes the functional connectivity of the network to construct a temporary task-relevant circuit for WM-guided behaviour (see also [27]).

According to the original computational model [23], an activity-silent coding scheme is more efficient than persistent firing models. Persistent firing could be energetically expensive, especially if WM is in near-constant use constructing and maintaining an up-to-date world model of the contents of the environment and the rules that govern them. The brain consumes about 20% of total energy and it is estimated that action potentials and postsynaptic effects of glutamate account for much of this expenditure (47% and 34%, respectively [28]). Therefore, neural economy places important constraints on ecologically optimal coding strategies [29]. For example, it has been estimated that less than 1% of neurons can be activated simultaneously [30]. It is difficult to rule out energy-efficient variants of persistent activity models of WM (e.g., WM codes could be sparsified across a cell population [31]). Nevertheless, a coding format that does not depend on an unbroken chain for persistent firing would have a clear cost advantage (i.e., sparsification in time).

WM coded in patterns of functional connectivity might also provide a neurobiologically plausible mechanistic account for WM-guided behaviour as a generalised state-dependent processing. Memories are not stored as an active representation but rather change the functional architecture of the neural network for future processing. As in long-term memory, WM is expressed by the way the network responds to new input. State-dependent readout would avoid the need for explicit mechanisms for comparing the internal representation with a separate representation reflecting the new input [23,32]. Moreover, because memories are stored in a format that is qualitatively different from perceptual representations, their informational content could be more resistant to interference from and/or confusion with other activity-based representations [33].

Despite considerable theoretical appeal, the synaptic model of WM is relatively difficult to test empirically. Extensive evidence confirms the prevalence of STSP [25] but its functional role has not yet been fully established. Microelectrode neurophysiology can only indirectly infer connectivity via interactions between simultaneous recordings. The gold-standard evidence for a monosynaptic connection is correlated spiking between neurons. In practice, however, the probability of sampling any two neurons with a measurable monosynaptic connection is extremely low (∼1–2% of all recorded pairs in [34]). Such a poor yield has been effectively prohibitive for testing synaptic plasticity within a standard primate neurophysiological study. Turning to the rodent model, researchers were able to collect sufficient samples of simultaneous recordings to show that the pattern of effective synaptic connections was dynamically modulated during a WM-dependent maze task [34] (Figure 2B). Large-scale studies in the primate PFC are required to provide more specific evidence for a synaptic theory of WM.

Synaptic plasticity is not the only candidate mechanism for a functional connectivity coding scheme for WM. For example, frequency-specific coherence could provide a complementary mechanism for rapid and temporary shifts in functional connectivity by phase aligning periods of excitability to maximise the opportunity for information transfer (and/or misaligning to suppress communication; communication through coherence [35]). Interestingly, researchers have recently reported frequency-specific coherence that dynamically established rule-specific subnetworks within the PFC [36] (Figure 2B). A similar mechanism for the rapid configuration of content-specific network architectures could be used to keep other forms of task-relevant material in WM [37].

## Dynamic coding for WM

**Figure 3:**
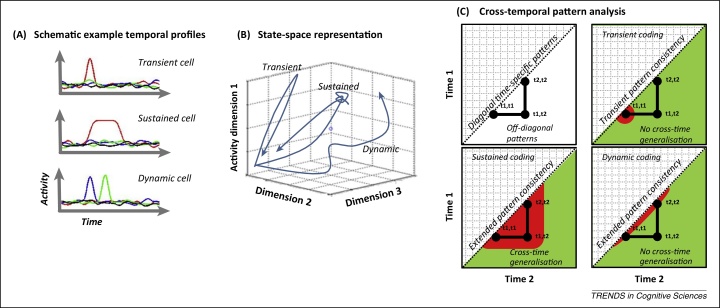
Representing dynamic population coding. **(A)** Schematic illustration of three different temporal coding profiles in three hypothetical cells: transient, sustained, and dynamic. Colours correspond to different experimental conditions (e.g., memoranda). **(B)** These dynamics can be visualised in a 3D state-space plot (for example, activity in three separate neurons). The trajectory through state space illustrates the dynamic profile. The trajectory for transient coding is illustrated as a simple path to a specific point in state space with immediate return. The sustained profile is also shown as a simple return trajectory, but with additional time spent at the target point in state space. Finally, dynamic coding is shown as a more complex path through state space. **(C)** The same dynamics can be computed for *n* dimensions (where *n* is the size of the neural population) and visualised using a 2D representation of cross-temporal pattern analysis. Essentially, this approach measures the consistency of a population code between time points (i.e., Time 1 along the y-axis, Time 2 along the x-axis). In this schematic, pattern consistency is colour coded red whereas nonconsistent patterns are colour coded green. Upper left panel illustrates the basic structure: diagonal values reflect time-specific patterns [*p*(*t*1,*t*1), *p*(*t*2,*t*2)]; off-diagonals reflect cross-temporal generalisation [*p*(*t*1,*t*2)]. Upper right panel illustrates the expected profile for transient coding, with reliable patterns at *p*(*t*1,*t*1) but not *p*(*t*2,*t*2). In the absence of coding at *t*2, it also follows that there is no cross-time generalisation at *p*(*t*1,*t*2). Lower left panel illustrates sustained coding, with consistent patterns at *p*(*t*1,*t*1) and *p*(*t*2,*t*2) but critically also significant cross-temporal generalisation *p*(*t*1,*t*2). This indicates a shared coding pattern at *t*1 and *t*2. By contrast, the lower right plot illustrates dynamic coding: consistent patterns at *p*(*t*1,*t*1) and *p*(*t*2,*t*2) but critically no significant cross-temporal generalisation [*p*(*t*1,*t*2)] (see also [39]).

Population decoding methods using a sliding temporal window on high-temporal-resolution data show that brain activity is highly dynamic [38,39] (summarised in Figure 3). Some of the most detailed studies of dynamic population coding have been performed in the olfactory system of the locust [40]. For example, specific odours trigger a series of activity patterns within antennal-lobe projection neurons [41]. This complex spatiotemporal activation pattern implies that population coding is time-specific: the same sensory information is represented over time, but in different spatial patterns that depend on the specific time point. Although the population eventually returned to a stable state, the most discriminative information for odour classification was observed along the most dynamic phase of the response. Downstream neurons that receive output signals from these projection neurons respond most vigorously during the most dynamic phase of the response. By contrast, activity is relatively mute in these putative readout neurons after the projection cells have returned to a relatively stable state.

Importantly, these dynamics are not specific to locust olfaction but are likely to constitute a general property of neural processing [38,100]. Of particular relevance here, dynamic population coding has also been observed in the monkey parietal cortex [42] and PFC [43] and could be especially important for WM-based decision making [44]. At the single-unit level, dynamic coding can be distinguished from transient or sustained firing by time-specific selectivity (Figure 3A). A dynamic cell might be engaged in processing across numerous time points (cf. transient cell-firing pattern) but the coding qualitatively depends on time during the processing window (cf. sustained firing pattern). Although it might be helpful to conceptualise distinct forms of temporal profile, these temporal characteristics vary along a continuum in the PFC rather than forming very distinct categories [45,46]. Such coding characteristics are best reflected in the full population response [46] rather than focusing on *a priori* defined categories of cell type [47].

At the population level, time dependency can be expressed as a dynamic trajectory through activity state space (Figure 3B), but this is difficult to visualise beyond three dimensions. Alternatively, time dependency can be expressed in a 2D cross-temporal pattern analysis (Figure 3C), where discriminative patterns at each time point are statistically compared with discriminative patterns at every other time point [39]. In this scheme, dynamic coding gives rise to a hallmark pattern of robust decoding along the within-time diagonal axis but little or no cross-generalisation between time points [39]. Although the same information is decodable from the ensemble activity (e.g., WM content), the underlying patterns of activity differ qualitatively over time.

**Figure 4:**
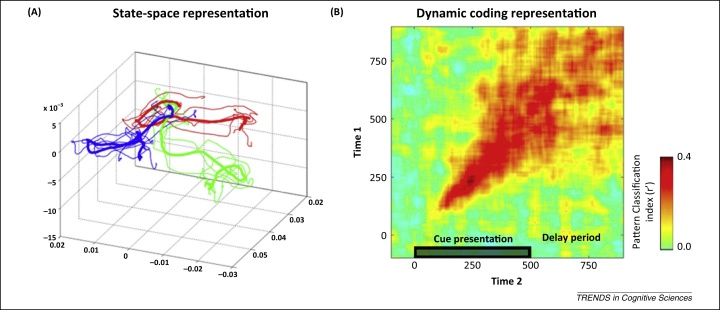
Evidence for dynamic coding of working memory (WM) in monkey prefrontal cortex (PFC). **(A)** The population-level geometric measures described in Figure 3 were applied in [44] to track WM coding in the monkey PFC. A state-space representation based on the dimensionality-reduced population response illustrates dynamic trajectories for each memory condition (three conditions, colour coded). **(B)** These dynamics can be seen clearly in the 2D cross-temporal analysis matrix, with robust WM-specific activity along the within-time diagonal axis and relatively poor temporal cross-generalisation at off-diagonal time points. Adapted from [44].

We previously observed hallmark features of dynamic coding in a delayed paired-associate task [44]. The initial response to a to-be-remembered memory item triggered a dynamic trajectory through activity state space (Figure 4A) resulting in strong time dependency (Figure 4B). Yet despite the spatiotemporal complexity of this dynamic code, information decodability peaked during the most energetic and dynamic phase before settling into a low-energy stable state during the delay period. Memory content could still be decoded during the delay period but the discriminative pattern did not resemble a straightforward continuation of the initial encoding of the information during the cue period. This contrasts with a simple form of a persistent coding in which activity patterns elicited by the presentation of task-relevant information are just clamped down and maintained throughout a memory delay. The discriminative pattern was also distinct from the population code for the expected target, in contrast to a simple preactivation form of prospective coding [48]. This pattern of dynamic coding is, however, consistent with a synaptic model of WM as outlined below.

Complex population dynamics can be predicted from the evolution of state-dependent processing in which the underlying response sensitivity of the network varies over time [38]. The response sensitivity, or hidden state, of a neural network is determined by the various neurophysiological parameters that determine how the network will respond to input at any given time point, especially functional connectivity (e.g., activity-dependent STSP). This neural state is ‘hidden’ simply because our recording techniques typically measure activity states only; that is, the response of the hidden state to input (extrinsic or intrinsic). Importantly, the hidden state is not stationary but is influenced by past experience, including long-term synaptic plasticity but also temporary changes in functional connectivity [25]. Temporal variability at the very shortest timescales means that the activity state is in constant flux due to a cascading interaction between hidden states and activity states. While input drives a specific response to a network according to the hidden state, the resultant activity state in turn reconfigures the hidden state. Therefore, the response to subsequent stimulation will trigger a unique response pattern according to the new hidden state. Moreover, this new pattern will further modulate the new hidden state of the system, thus determining the response to the next input, and so on [38]. The reciprocal interaction between the activity state and the hidden state results in a complex spatiotemporal trajectory through state space observed throughout different animal models and brain areas [49]. The full trajectory is reproducible across trials to the extent that the hidden state relaxes back to the baseline connective pattern after some time period. This baseline hidden state is determined by more stable connections established via long-term plasticity, whereas the time constants that influence temporary changes in the hidden state determine the duration of activity-silent WM. Maintenance for longer durations presumably requires periodic refreshing; for example, via intrinsic oscillations [23,50] or more explicit attention-related rehearsal [51].

## Revealing the ‘silent’ WMs

**Figure 5:**
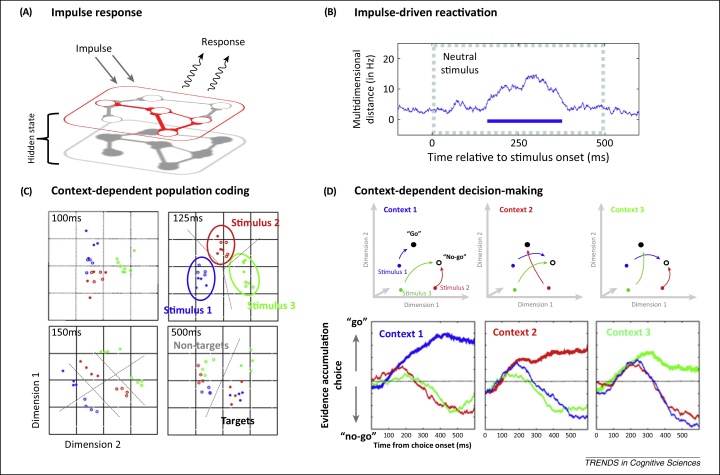
Context-dependent readout for ‘activity-silent’ working memory (WM). **(A)** Synaptic WM is effectivity activity silent; however, the information content can be inferred from a shift in the behaviour of the network. This can be probed using an impulse of activity to drive the network (Box 1). **(B)** We previously observed evidence for impulse-driven reactivation of WM in the prefrontal cortex. Using multivariate statistics, we found that a neutral stimulus triggered a distinct pattern of activity that reflected the content of WM (blue trace) although the driving stimulus was exactly the same for all WM conditions (from [44]). **(C)** A temporary shift in the response profile of the network could also provide a basis for context-dependent processing during WM-guided behaviour. For example, we found that during WM-guided behaviour population-level activity states quickly evolved from representing the physical properties of the choice stimuli (at 125 ms; stimuli are coloured coded) to the decision-relevant coding from ∼150 ms (i.e., target versus non-target; from [44]). **(D)** This process can be schematised as a context-dependent path through activity state space. A functional shift in the hidden state effectively conditions the context-dependent input/output behaviour of the network to map stimulus-specific activity states to the context-relevant position in state space (upper panel; from [44]). This flexible mapping could be framed as a context-dependent decision process. In the lower panel, the results from (C) are replotted as the accumulation of evidence for the ‘go’ or ‘no-go’ response as a function of the current rules (i.e., if context 1, then evidence for stimulus 1 supports a ‘go’ response, but stimuli 2 and 3 provide counter-evidence; upper panel adapted from [44]).

Although hidden states are effectively activity silent, their structure can be inferred from the input–output behaviour of the network (i.e., state-dependent response; see Figure 5). This is a basic readout mechanism proposed for synaptic WM [23,32] but also predicts that any activity drive to the network should elicit a state-dependent response that reflects the content in WM. Consider an example of active sensing (e.g., sonar), where a simple impulse (e.g., ‘ping’) is used to probe the hidden contours of an unseen structure (Figure 5A). In the same manner, the impulse response to a neural perturbation faithfully reflects the current connectivity architecture that determines input–output mapping of the neural network. If the impulse is held constant over trials within a WM condition, any WM-related difference in output can be attributed to a change in the state of the system, including hidden states. Consistent with this argument, we previously found that a task-irrelevant stimulus presented during a WM delay triggered a population-level response in the PFC that clearly differentiated between different content in WM [44] (Figure 5B).

In basic information terms, a content-specific shift in hidden state could be decoded from the resultant change in the network response profile. However, synaptic WM could also provide a more explicit context-dependent mapping for WM-guided behaviour [44,52]. Previously, we found that population-level tuning profiles in the PFC rapidly adapt to accommodate changes in behavioural context [44]. The transition from stimulus-specific representations to context-dependent coding could be visualised in 2D space using multidimensional scaling (Figure 5C; see also [53]). At this decision stage, perceptually distinct choice stimuli (colour coded) could be either a current target (filled circles) or a distractor (unfilled circles) depending on the trial context. The initial separation in state space at around 100–125 ms differentiated the neural response as a function of stimulus identity (stimulus specificity). However, by 150 ms there was already clear evidence for separation by decision value: target versus non-target. Importantly, context-dependent separation was not arbitrary, as a common decision boundary separated target from non-target irrespective of stimulus type. This implies systematic routing of stimulus-specific patterns to task-appropriate positions in state space (Figure 5D, upper panel), enabling evidence accumulation according to a flexible decision rule (Figure 5D, lower panel).

## Implications for cognitive models

Cognitive studies demonstrate that multiple items held concurrently in WM are not all necessarily represented in the same way [54–57]. For example, a recent multistate model proposes that only a single item within WM is held in a prioritised active state [33]. This ‘priority item’ is also the current focus of attention and automatically biases processing in favour of matching input (e.g., [58]). This is equivalent to the search template in the biased competition model of attention [59–61], but also allows other, less-relevant items to remain effectively dormant (e.g., activity silent). Importantly, prioritisation is flexible: as items become more or less relevant to behaviour, they can become more or less active accordingly [54]. Retrospective cueing studies provide empirical evidence for shifting priorities within WM [62,63], with specific benefits on WM readout [64]. Brain-imaging studies of retro-cueing provide evidence that attended items in WM are associated with discriminable patterns of neural activity, whereas unattended items are effectively activity silent although they can be accurately recalled at the end of the delay period [65].

Recent evidence from human electroencephalography (EEG) suggests that an active form of WM is especially important for flexible moment-to-moment shifts in attentional set. For example, contralateral delay activity (a putative EEG marker of WM) is evident when WM is first used to maintain a search template but diminishes when the same attentional template is used for several consecutive trials [66]. This could reflect a transfer to silent WM as a function of learning [67]. Similar to the monkey neurophysiology, these studies imply that although activity during memory delays might serve an important role in prioritising mnemonic content in preparation for efficient WM-guided behaviour, persistent activity is not necessary for maintenance *per se*. Activity-silent representations can still support WM-guided behaviour.

The dynamic coding perspective outlined in this review could have important implications for understanding capacity limits in WM. For example, dynamic coding would predict that capacity limits are more closely tied to limits in encoding [68,69] and/or readout [70] rather than ongoing competition between active representations during the delay period [71]. It will be important to relate dynamic coding to limits in the amount [72] and quality of information in WM [73] or how information is bound between features [74,75]. Individual differences in encoding/readout could also help explain the relationship between WM and general intelligence [76]. At first glance, it would seem intuitive that a larger WM storage capacity should provide more computational power for supporting abstract reasoning and general problem solving. However, individual-difference studies suggest that selection [69] and/or object-based grouping strategies [77] appear to mediate the WM–IQ relationship rather than raw storage capacity. Similarly, cognitive studies of goal neglect suggest that low-performing participants systematically fail to use behaviourally relevant information provided at initial task instruction [78,79], although they can accurately recall the neglected rules at the end of the experiment. Goal neglect implies a clear distinction between maintenance and use: holding task-relevant information in mind might be necessary but not sufficient for WM-guided behaviour.

On a more practical level, activity-silent WM poses a challenge to research in cognitive neuroscience. Our basic tools are designed to measure activity states, but clearly we need to develop new ways to measure activity-silent states that might be important for WM. This could include functional connectivity measures (statistical dependencies between simultaneous recordings), but also perturbation methods to infer changes in response profile (e.g., Figure 5A). If we consider active neural states only, we risk ignoring the broader landscape of temporary neural states that mediate flexible context-dependent processing.

This review has focused on how dynamic coding might contribute to WM-guided behaviour, but the same framework could also help to explain more general cognitive control, including context-dependent decision making [44] or attentional control [80]. Essentially, dynamic coding reconsiders WM as the current response potential of a network, conditioned by the recent stimulation history that determines the behavioural context. In this sense, standard WM tasks are reframed as context-dependent decision-making tasks [52,81], wherein the memory stimulus defines the stimulus–response mapping to the probe (e.g., match versus non-match). Instead of maintaining a perceptual representation *per se*, dynamic coding maintains the behaviourally relevant rule. Classic models of cognitive control suggest that active states in the PFC encoding current task context modulate other brain areas to reconfigure stimulus–response mapping for rule-guided behaviour [82]; however, a dynamic coding framework essentially proposes that this remapping could be instantiated more directly to mediate context-dependent behaviour (akin to ultra-rapid adaptive coding [83] or task-specific functional ensembles [84]). It will be important for future research to disentangle the relative contributions of stationary activity states representing context [85] and functional connectivity in context-dependent processing.

Dynamic coding is also conceptually related to mixed selectivity [45,46], which effectively maximises the dimensionality of a network. Time specificity effectively adds another dimension to the coding potential of a network [49]. Indeed, the coding potential is directly proportional to the independence between time points. This is expressed in the off-diagonal correlations of the cross-temporal analyses. Nonlinear activity dynamics could also mediate context-dependent processing. For example, recurrent networks can learn to accumulate evidence according to an independent context signal [85]. Recurrent dynamics could also mediate hysteresis for memories (e.g., [86]) and dynamical systems analyses show that time-specific activity states could help energise subsequent dynamics [87]. It will be important to integrate known mechanisms for short-term synaptic plasticity [25] into such models to evaluate the relative contribution of various potential processes for mediating context-dependent processing in cortical circuits.

## A unique role for the PFC

Dynamic coding is almost certainly not limited to the PFC but is observed from locust olfaction to the highest level of the primate cortex. What makes dynamic coding in the PFC so important for WM? The intrinsic time constants of PFC neurophysiology could provide extended hysteresis sufficient for WM [26,88]. However, perhaps more importantly, the PFC occupies an ideal network position [89,90] to exploit a general principle of dynamic coding for WM in the service of flexible cognition.

Diverse input connections deliver information about the external environment derived from the senses along with internal variables such as reward values, abstract rules, and long-term goals [89]. At the apex of the feedforward hierarchy, information that reaches the PFC is already highly preprocessed, filtered, and integrated for task relevance [91]. Therefore, information that drives changes to the PFC is already likely to be task relevant and optimised for cognitive control [89,90]. By contrast, equivalent forms of dynamic coding in lower brain areas [32] such as the perceptual cortex [92] could reflect ‘lower’ forms of memory (e.g., iconic memory [93]). Moreover, because perceptual areas are constantly updated by new input, temporary state changes may be particularly vulnerable to overwriting, thus limiting the durability and lifespan of non-prefrontal memory traces [94]. The functional insulation of the PFC from moment-to-moment fluctuations in irrelevant signals provides an ideal space for maintaining information necessary for complex time-extended behaviour [95]. Finally, extensive output connections also place the PFC in an ideal neuroanatomical position to modulate processing in other brain areas according to the product of external and internal variables [89,90].

## Concluding remarks

**Figure 6:**
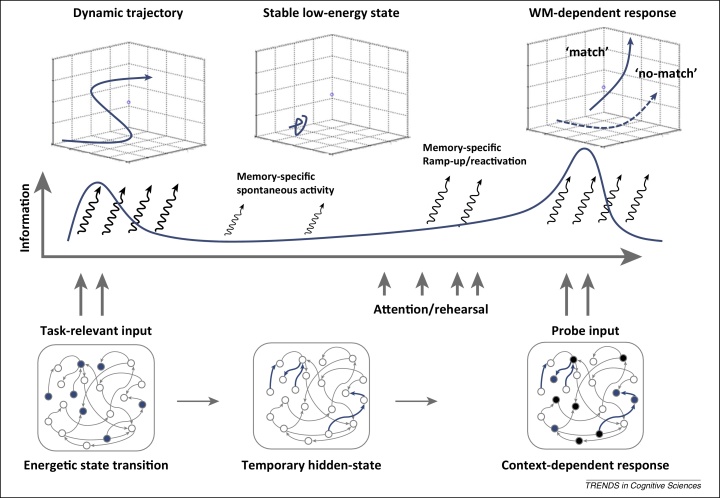
Summary schematic of dynamic coding for working memory (WM)-dependent behaviour. The initial input triggers a specific pattern in activity state that in turn alters the underlying hidden state of the network via a temporary shift in functional connectivity (e.g., short-term synaptic plasticity [25], or coherence [35]). Activity-dependent changes in hidden state drive a dynamic trajectory during the initial high-energy phase via the reciprocal interaction between hidden states and the activity states that modulate them [43,44]. After activity has relaxed to baseline levels, the hidden state remains patterned according to the WM item. Although in principle this temporary hidden state could be ‘activity silent’, any spontaneous activity in the network will be patterned according to the WM context, resulting in a WM-specific activity state during spontaneous firing [44]. This kind of ‘baseline emission’ could help explain the content-specific delay activity observed under some circumstances [32]. Increasing the level of network activity via attention/rehearsal mechanisms could increase the discriminability of the activity state, resulting in ‘ramp-up’ delay activity [19,20] or task-dependent ‘reactivations’ [22]. Finally, when the critical memory cue is presented, the context-dependent response maps activity states for WM-guided behaviour (e.g., match/non-match decision; see [44]).

A dynamic coding framework for WM is summarised in Figure 6. This model effectively recasts WM as a flexible decision process where the memory item defines a temporary decision circuit for accumulating evidence during WM-dependent processing (e.g., match/non-match). The same framework could be applied for more general forms of context-dependent processing [38], including rule-dependent processing in the PFC [44]. This shift in network input/output behaviour could provide a neurophysiological basis for a form of ultra-rapid adaptive coding [83] that would be suitable for trial-wise assignment of neuronal selectivity in the PFC. Coding in the PFC could adapt as rapidly as thought itself.

This review identifies a number of outstanding questions (Box 2). However, it is important to note that future advances will depend critically on new methodological approaches for characterising a diversity of neural states. Population-level analyses provide a powerful set of tools for characterising subtle patterns of neural activity as trajectories through multidimensional state space. However, even these activity-state representations provide only a partial glimpse of the neural states that underlie cognition. New methods to measure the consequences of experimentally controlled perturbations of the neural state indicate a promising avenue for future research (Box 1). Future advances also critically depend on bridging levels of analysis. This review has focused on neurophysiological evidence from nonhuman primates. Ultimately, we must find new ways to measure and interpret dynamic neural states in the human brain. This is particularly important for studying high-level cognition such as WM. The nonhuman primate is an excellent model, but even monkey behaviour displays only a fraction of the cognitive flexibility that defines the human brain. Moreover, without the benefit of verbal instruction, animal experiments require extensive training regimens based on reinforcement leaning. The burden of learning further diminishes the nature of flexibility in monkey studies. Future progress therefore depends on better methods to bridge levels of analysis, from high-precision invasive recordings to large-scale dynamics in noninvasive neuroimaging. This is essential if we are to translate insights gained in our primate cousins to the target system: human cognition.

## Figures and Tables

**Figure I:**
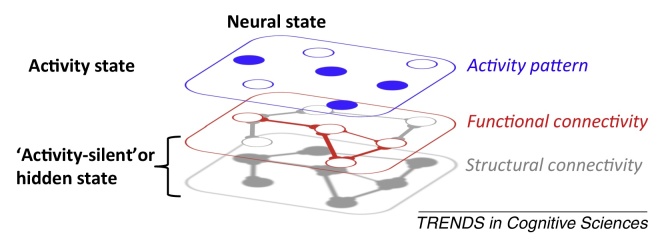
Schematic of layered neural states. The neural state comprises the ‘activity state’ that is measured in typical experiments, but also ‘hidden states’ such as functional connectivity and structural connectivity. Short-term changes in these hidden states could play an important role in high-level cognition, including working memory.

## Box 1 Inferring hidden neural states

Activity-silent representations pose an obvious challenge for contemporary neuroscience, which is dominated by measurement and analysis of neural activity. It is perhaps unsurprising that persistent activity models have dominated cognitive neuroscience. Persistent activity is simply easy to measure. It is critical therefore that we develop new tools for measuring a wider diversity of neural states, including hidden states (Figure I). Here are some promising avenues.

**Intrinsic network dynamics** can provide a window on the current hidden state of a network [38]. In this respect, hidden states should have their own signature hum. Using sensitive population-level analyses, it may be possible to detect subtle differences in activity patterns associated with spontaneous fluctuations in activity. It has been argued that previous evidence for persistent delay activity could be attributed to a passive emission of the WM hidden state in the visual system [32]. A similar signature should also be evident in the correlational structure of activity. Measures of functional connectivity should be especially sensitive if WM is directly encoded in temporary shifts of the underlying network architecture. However, these measures of intrinsic dynamics critically depend on sufficient spontaneous activity. This can be especially problematic for measures of spiking activity, which can be temporally sparse in the PFC (∼1 Hz).

**Driven network dynamics** can also provide an important window on the hidden state. Rather than simply measuring the structure of spontaneous activity, the hidden state can be probed using a more forceful perturbation approach. For example, a driving sensory stimulus can increase the overall energy in a neural network to amplify the idiosyncratic emission of the hidden state [44]. The network could also be perturbed more directly using an experimentally controlled injection of current (e.g., microelectrode stimulation, transcranial magnetic stimulation, optogenetics). These perturb-and-measure approaches would also yield second-order statistics, such as the correlation or coherence between nodes during the evoked response.

These concepts are not limited to local activity patterns and connectivity profiles but can also apply to long-range network connectivity to reveal more global-network hidden states. Moreover, these tools could be useful in a broad domain of cognitive neuroscience. The activity states that we usually measure provide only an incomplete picture of the diversity of neural states underlying cognition. This might be especially true for more tonic cognitive states such as WM, attentional state, and task set. The ultimate success of future research will depend on new approaches to existing measurement techniques to probe diverse neural states, including activity-silent states.

## Box 2 Outstanding questions

• What are the neurophysiological principles underlying dyanmic coding? The framework outlined in this review assumes only a rapid and temporary shift in the response profile of the neural population. We have focused on short-term synaptic plasticity as an attractive candidate; however, future research should verify its role in WM and also consider other, complementary and/or alternative mechanisms such as frequency-specific coherence [36] or activity-dependent network attractors [85,87].
• What is the role of other brain areas? The importance of the PFC in cognitive control is well established, yet dynamic coding is likely to be a general phenomenon of neural networks across the cortical heirarchy. Dynamic coding in the PFC could be especially important for WM and cognitive control because of the more global network position of the PFC. However, the PFC is increasingly considered part of a network of high-level brain areas [96] working in close coordination with the parietal cortex [97]. Future work should clarify how dynamic coding is coordinated across the broader network of brain areas for high-level cognition.
• Capacity limits are a key feature of WM [98] and might play an important role in setting more general limits in high-level cognition [77]. How can a dynamic coding framework help explain cognitive capacity limits in general? Could a synaptic model of WM specifically account for limits in the amount and quality of information in WM [72,73]?
• Is there a continuity of time frames for dynamic coding? Can a single principle be applied from iconic memory to WM [93] and ultimately long-term memory [70]?
• Critically, WM must be robust to distraction. This raises the important question of whether dynamic coding can be selective for to-be-encoded input, or is this hidden state altered by any input activity?
• Finally, it will be important for future research to consider the potential role of specific neurotransmitter systems such as dopamine for gating WM encoding [99]. How do neuromodulators contribute to hidden states within a dynamic coding framework?

## Glossary

Activity state space: an *n*-dimensional representation in which each axis is defined by neural activity along each dimension (i.e., cells). Often, activity-state-space representations are reduced to two or three dimensions for visualisation purposes using a dimensionality reduction method such as principal component analysis. However, analyses can be readily performed in the full dimensionality using basic geometry.
Calcium kinetics: a possible mechanism for temporary synaptic facilitation. The synaptic model of WM proposes that residual calcium at synaptic terminals serves as a memory ‘buffer’ following content-specific excitation. Critically, the synaptic calcium kinetics has a relatively long time constant, allowing an activity-silent synaptic trace to span approximately 1 s.
Communication through coherence: a general principle that functional connectivity can be modulated by neuronal synchrony. Activity of neural populations tends to oscillate, resulting in periodic fluctuations in excitability. If neural populations oscillate at the same frequency, periods of excitability can be phase aligned to maximise the opportunity for information transfer and/or misaligned to suppresses communication.
Content-specific delay activity: neural activity during a memory delay period that varies as a function of the content of WM. For example, some cells might be more active during maintenance of memory item ‘A’ whereas others might fire more for memory item ‘B’. Content-specific delay activity is often considered the hallmark of a WM representation.
Functional connectivity: a measure of the relationship between activation time series at two recording sites. Functional connectivity can be quantified as the statistical dependency between anatomically separable simultaneous recordings and is thought to reflect functional coupling between areas.
Hidden states: a relatively nonspecific term for the various neurophysiological parameters that determine the response sensitivity of a neural network, such as synaptic efficacy, membrane potentials, and neurotransmitter concentrations. They are hidden simply because they are not directly observable by standard recording methods. Although the activity state of a network is usually the focus of research, the behaviour of a network critically depends on the current state of these underlying parameters.
Hysteresis: the extent to which a system is dependent on both the current state and past states. Hysteresis is an important concept in a range of fields, from engineering to biology and economics.
Sparse coding: an efficient coding scheme that draws on only a subset of neurons. However, when considering temporally extended representations (i.e., WM) it also makes sense to consider sparsity in time. An efficient coding scheme would result in relatively few action potentials over time.

## References

[1] Goldman-Rakic P.S. (1995). Cellular basis of working memory. Neuron.

[2] Butters N., Pandya D. (1969). Retention of delayed-alternation: effect of selective lesions of sulcus principalis. Science.

[3] Stamm J.S. (1969). Electrical stimulation of monkeys’ prefrontal cortex during delayed-response performance. J. Comp. Physiol. Psychol..

[4] Pasternak T., Greenlee M.W. (2005). Working memory in primate sensory systems. Nat. Rev. Neurosci..

[5] Postle B.R., Jolicoeur P. (2015). Neural bases of the short-term retention of visual information. Mechanisms of Sensory Working Memory: Attention and Performance XXV.

[6] Zaksas D., Pasternak T. (2006). Directional signals in the prefrontal cortex and in area MT during a working memory for visual motion task. J. Neurosci..

[7] Miller E.K. (1993). Activity of neurons in anterior inferior temporal cortex during a short-term memory task. J. Neurosci..

[8] van de Ven V. (2012). Topographic contribution of early visual cortex to short-term memory consolidation: a transcranial magnetic stimulation study. J. Neurosci..

[9] Fuster J.M., Alexander G.E. (1971). Neuron activity related to short-term memory. Science.

[10] Kubota K., Niki H. (1971). Prefrontal cortical unit activity and delayed alternation performance in monkeys. J. Neurophysiol..

[11] Funahashi S. (1989). Mnemonic coding of visual space in the monkey's dorsolateral prefrontal cortex. J. Neurophysiol..

[12] Miller E.K. (1996). Neural mechanisms of visual working memory in prefrontal cortex of the macaque. J. Neurosci..

[13] Curtis C.E., D’Esposito M. (2003). Persistent activity in the prefrontal cortex during working memory. Trends Cogn. Sci..

[14] Wang X.J. (2001). Synaptic reverberation underlying mnemonic persistent activity. Trends Neurosci..

[15] Durstewitz D. (2000). Neurocomputational models of working memory. Nat. Neurosci..

[16] Compte A. (2000). Synaptic mechanisms and network dynamics underlying spatial working memory in a cortical network model. Cereb. Cortex.

[17] Sreenivasan K.K. (2014). Revisiting the role of persistent neural activity during working memory. Trends Cogn. Sci..

[18] Shafi M. (2007). Variability in neuronal activity in primate cortex during working memory tasks. Neuroscience.

[19] Watanabe K., Funahashi S. (2007). Prefrontal delay-period activity reflects the decision process of a saccade direction during a free-choice ODR task. Cereb. Cortex.

[20] Barak O. (2010). Neuronal population coding of parametric working memory. J. Neurosci..

[21] Nobre A.C. (2007). The hazards of time. Curr. Opin. Neurobiol..

[22] Watanabe K., Funahashi S. (2014). Neural mechanisms of dual-task interference and cognitive capacity limitation in the prefrontal cortex. Nat. Neurosci..

[23] Mongillo G. (2008). Synaptic theory of working memory. Science.

[24] Barak O., Tsodyks M. (2014). Working models of working memory. Curr. Opin. Neurobiol..

[25] Zucker R.S., Regehr W.G. (2002). Short-term synaptic plasticity. Annu. Rev. Physiol..

[26] Bernacchia A. (2011). A reservoir of time constants for memory traces in cortical neurons. Nat. Neurosci..

[27] Erickson M.A. (2010). A single brief burst induces GluR1-dependent associative short-term potentiation: a potential mechanism for short-term memory. J. Cogn. Neurosci..

[28] Attwell D., Laughlin S.B. (2001). An energy budget for signaling in the grey matter of the brain. J. Cereb. Blood Flow Metab..

[29] Laughlin S.B. (2001). Energy as a constraint on the coding and processing of sensory information. Curr. Opin. Neurobiol..

[30] Lennie P. (2003). The cost of cortical computation. Curr. Biol..

[31] Woloszyn L., Sheinberg D.L. (2009). Neural dynamics in inferior temporal cortex during a visual working memory task. J. Neurosci..

[32] Sugase-Miyamoto Y. (2008). Short-term memory trace in rapidly adapting synapses of inferior temporal cortex. PLoS Comput. Biol..

[33] Olivers C.N. (2011). Different states in visual working memory: when it guides attention and when it does not. Trends Cogn. Sci..

[34] Fujisawa S. (2008). Behavior-dependent short-term assembly dynamics in the medial prefrontal cortex. Nat. Neurosci..

[35] Fries P. (2005). A mechanism for cognitive dynamics: neuronal communication through neuronal coherence. Trends Cogn. Sci..

[36] Buschman T.J. (2012). Synchronous oscillatory neural ensembles for rules in the prefrontal cortex. Neuron.

[37] Salazar R.F. (2012). Content-specific fronto-parietal synchronization during visual working memory. Science.

[38] Buonomano D.V., Maass W. (2009). State-dependent computations: spatiotemporal processing in cortical networks. Nat. Rev. Neurosci..

[39] King J.R., Dehaene S. (2014). Characterizing the dynamics of mental representations: the temporal generalization method. Trends Cogn. Sci..

[40] Laurent G. (2002). Olfactory network dynamics and the coding of multidimensional signals. Nat. Rev. Neurosci..

[41] Mazor O., Laurent G. (2005). Transient dynamics versus fixed points in odor representations by locust antennal lobe projection neurons. Neuron.

[42] Crowe D.A. (2010). Rapid sequences of population activity patterns dynamically encode task-critical spatial information in parietal cortex. J. Neurosci..

[43] Meyers E.M. (2008). Dynamic population coding of category information in inferior temporal and prefrontal cortex. J. Neurophysiol..

[44] Stokes M.G. (2013). Dynamic coding for cognitive control in prefrontal cortex. Neuron.

[45] Rigotti M. (2013). The importance of mixed selectivity in complex cognitive tasks. Nature.

[46] Miller E.K., Fusi S. (2013). Limber neurons for a nimble mind. Neuron.

[47] Raposo D. (2014). A category-free neural population supports evolving demands during decision-making. Nat. Neurosci..

[48] Rainer G. (1999). Prospective coding for objects in primate prefrontal cortex. J. Neurosci..

[49] Stokes M. (2011). The spatiotemporal structure of population coding in monkey parietal cortex. J. Neurosci..

[50] Lisman J.E., Idiart M.A. (1995). Storage of 7 ± 2 short-term memories in oscillatory subcycles. Science.

[51] Awh E. (1998). Rehearsal in spatial working memory. J. Exp. Psychol. Hum. Percept. Perform..

[52] Deco G. (2010). Synaptic dynamics and decision making. Proc. Natl. Acad. Sci. U.S.A..

[53] Kriegeskorte N., Kievit R.A. (2013). Representational geometry: integrating cognition, computation, and the brain. Trends Cogn. Sci..

[54] Oberauer K. (2002). Access to information in working memory: exploring the focus of attention. J. Exp. Psychol. Learn. Mem. Cogn..

[55] Larocque J.J. (2014). Multiple neural states of representation in short-term memory? It's a matter of attention. Front. Hum. Neurosci..

[56] Nee D.E., Jonides J. (2013). Trisecting representational states in short-term memory. Front. Hum. Neurosci..

[57] Zokaei N. (2014). Causal evidence for a privileged working memory state in early visual cortex. J. Neurosci..

[58] Soto D. (2010). Working memory enhances visual perception: evidence from signal detection analysis. J. Exp. Psychol. Learn. Mem. Cogn..

[59] Desimone R., Duncan J. (1995). Neural mechanisms of selective visual attention. Annu. Rev. Neurosci..

[60] Chelazzi L. (1993). A neural basis for visual search in inferior temporal cortex. Nature.

[61] Stokes M. (2009). Shape-specific preparatory activity mediates attention to targets in human visual cortex. Proc. Natl. Acad. Sci. U.S.A..

[62] Griffin I.C., Nobre A.C. (2003). Orienting attention to locations in internal representations. J. Cogn. Neurosci..

[63] Landman R. (2003). Large capacity storage of integrated objects before change blindness. Vision Res..

[64] Murray A.M. (2013). Attention restores discrete items to visual short-term memory. Psychol. Sci..

[65] Lewis-Peacock J.A. (2012). Neural evidence for a distinction between short-term memory and the focus of attention. J. Cogn. Neurosci..

[66] Carlisle N.B. (2011). Attentional templates in visual working memory. J. Neurosci..

[67] Gunseli E. (2014). Effects of search difficulty on the selection, maintenance, and learning of attentional templates. J. Cogn. Neurosci..

[68] Tsubomi H. (2013). Neural limits to representing objects still within view. J. Neurosci..

[69] Linke A.C. (2011). Encoding strategy accounts for individual differences in change detection measures of VSTM. Neuropsychologia.

[70] Nairne J.S. (2002). Remembering over the short-term: the case against the standard model. Annu. Rev. Psychol..

[71] Edin F. (2009). Mechanism for top-down control of working memory capacity. Proc. Natl. Acad. Sci. U.S.A..

[72] Zhang W., Luck S.J. (2008). Discrete fixed-resolution representations in visual working memory. Nature.

[73] Bays P.M., Husain M. (2008). Dynamic shifts of limited working memory resources in human vision. Science.

[74] Wheeler M.E., Treisman A.M. (2002). Binding in short-term visual memory. J. Exp. Psychol. Gen..

[75] Bays P.M. (2011). Storage and binding of object features in visual working memory. Neuropsychologia.

[76] Conway A.R. (2003). Working memory capacity and its relation to general intelligence. Trends Cogn. Sci..

[77] Fukuda K. (2010). Quantity, not quality: the relationship between fluid intelligence and working memory capacity. Psychon. Bull. Rev..

[78] Duncan J. (2008). Goal neglect and Spearman's g: competing parts of a complex task. J. Exp. Psychol. Gen..

[79] Bhandari A., Duncan J. (2014). Goal neglect and knowledge chunking in the construction of novel behaviour. Cognition.

[80] Astrand E. (2015). Differential dynamics of spatial attention, position, and color coding within the parietofrontal network. J. Neurosci..

[81] Pearson B. (2014). Working memory retrieval as a decision process. J. Vis..

[82] Miller E.K. (2000). The prefrontal cortex and cognitive control. Nat. Rev. Neurosci..

[83] Duncan J. (2001). An adaptive coding model of neural function in prefrontal cortex. Nat. Rev. Neurosci..

[84] Buzsaki G. (2010). Neural syntax: cell assemblies, synapsembles, and readers. Neuron.

[85] Mante V. (2013). Context-dependent computation by recurrent dynamics in prefrontal cortex. Nature.

[86] Maass W. (2002). Real-time computing without stable states: a new framework for neural computation based on perturbations. Neural Comput..

[87] Kaufman M.T. (2014). Cortical activity in the null space: permitting preparation without movement. Nat. Neurosci..

[88] Murray J.D. (2014). A hierarchy of intrinsic timescales across primate cortex. Nat. Neurosci..

[89] Fuster J.M. (1997). The Prefrontal Cortex: Anatomy, Physiology, and Neuropsychology of the Frontal Lobe.

[90] Miller E.K., Cohen J.D. (2001). An integrative theory of prefrontal cortex function. Annu. Rev. Neurosci..

[91] Passingham D., Sakai K. (2004). The prefrontal cortex and working memory: physiology and brain imaging. Curr. Opin. Neurobiol..

[92] Nikolic D. (2009). Distributed fading memory for stimulus properties in the primary visual cortex. PLoS Biol..

[93] Jaaskelainen I.P. (2011). Short-term plasticity as a neural mechanism supporting memory and attentional functions. Brain Res..

[94] Sligte I.G. (2008). Are there multiple visual short-term memory stores?. PLoS ONE.

[95] Fuster J.M. (2001). The prefrontal cortex – an update: time is of the essence. Neuron.

[96] Dosenbach N. (2006). A core system for the implementation of task sets. Neuron.

[97] Duncan J. (2010). The multiple-demand (MD) system of the primate brain: mental programs for intelligent behaviour. Trends Cogn. Sci..

[98] Buschman T.J. (2011). Neural substrates of cognitive capacity limitations. Proc. Natl. Acad. Sci. U.S.A..

[99] Hazy T.E. (2007). Towards an executive without a homunculus: computational models of the prefrontal cortex/basal ganglia system. Philos. Trans. R. Soc. Lond. B: Biol. Sci..

[100] Goel A., Buonomano D.V. (2014). Timing as an intrinsic property of neural networks: evidence from in vivo and in vitro experiments. Philos. Trans. R. Soc. Lond. B: Biol. Sci..

